# Supplementary material for: Tumor MHC Expression Guides First-Line Immunotherapy Selection in Melanoma
Source: Cancers (Basel). 2020 Nov 14;12(11):3374. doi: 10.3390/cancers12113374 (PMC7696726; doi:10.3390/cancers12113374)
Supplement: Supplementary file 1 [file cancers-12-03374-s001.pdf]

Supplementary Material

Tumor MHC Expression Guides First-Line Immunotherapy Selection in Melanoma

Elena Shklovskaya, Jenny H Lee, Su Yin Lim, Ashleigh Stewart, Bernadette Pedersen, Peter Ferguson, Robyn PM Saw, John F Thompson, Brindha Shivalingam, Matteo S Carlino, Richard A Scolyer, Alexander M Menzies, Georgina V Long, Richard F Kefford and Helen Rizos

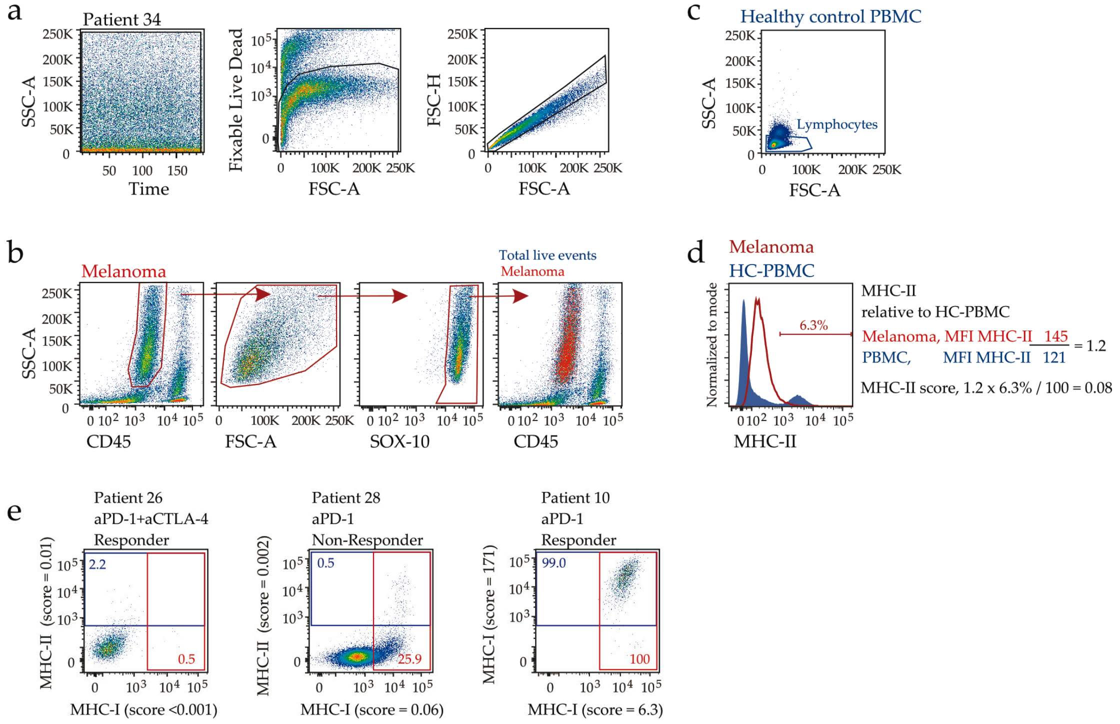

Figure S1. Flow cytometric analysis of melanoma cells in enzymatically dissociated tumor biopsies. (**a**) General gating (left to right): time gate, live cell gate, single cell gate. (**b**) Melanoma cell gating (left to right): CD45-negative, side scatter (SSC-A) high; forward scatter (FCS-A) high; SOX-10 positive; overlay of SOX-10 positive melanoma cells (red) onto total live events (pseudocolor plot). (**c**) Healthy control (HC) PBMC lymphocyte gating (SSC-A and FCS-A low). (**d**) MHC-II expression on tumor cells (red histogram) and control PBMC (blue shaded histogram). Fraction of melanoma cells expressing the marker is indicated. Calculations for melanoma MHC-II expression relative to HC-PBMC, and MHC-II expression score are shown (MFI, geometric mean fluorescent intensity). (**e**) Representative examples of MHC expression in three tumors, with frequency of positive (MHC-I, red; MHC-II, blue) and MHC expression scores indicated. Patient ID, therapy and treatment response are indicated above the plots.

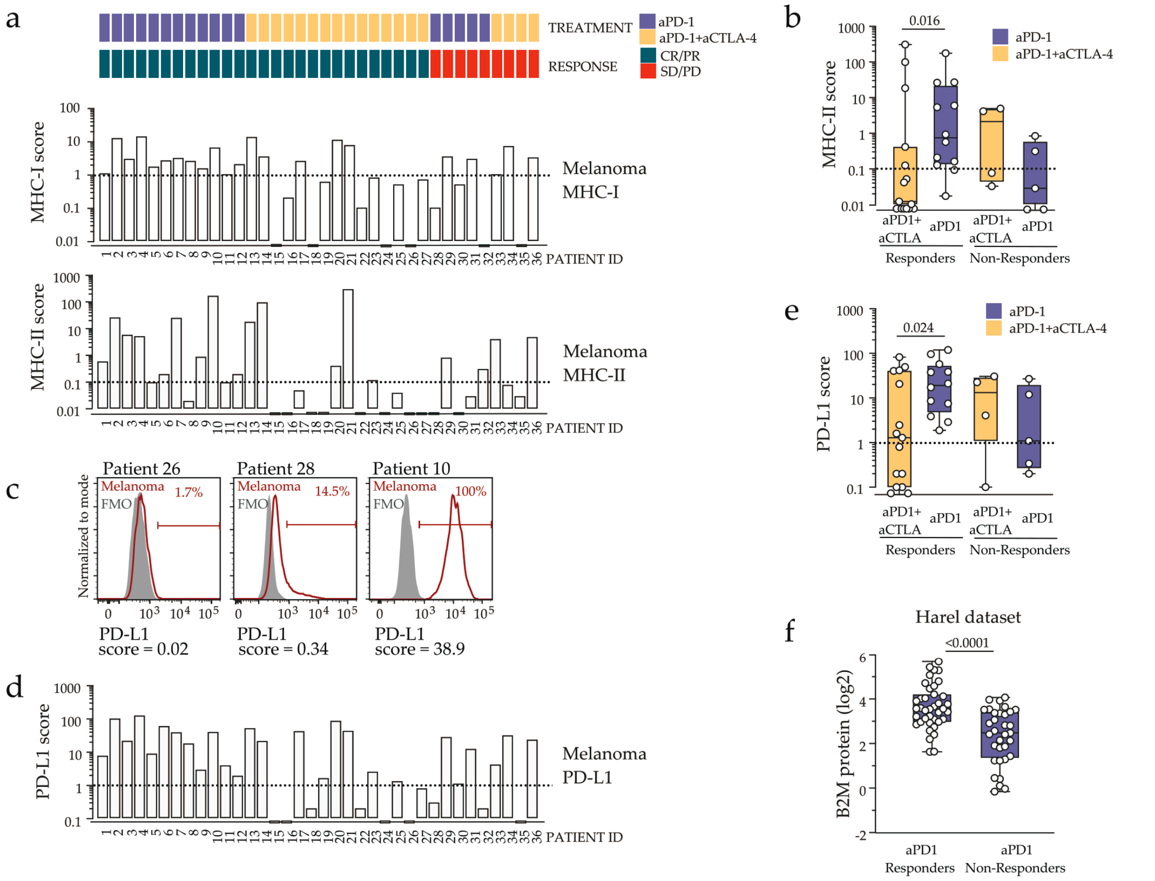

Figure S2. Flow cytometric evaluation of tumor MHC-I, MHC-II and PD-L1 expression in pretreatment biopsies. (**a**) Melanoma MHC-I and MHC-II expression scores for the 36 samples included in the study. Treatment, response status and patient ID are indicated. Dotted lines mark expression thresholds. (**b**) Melanoma MHC-II expression score stratified by therapy and patient response. Boxes show the interquartile range and the median, with Mann-Whitney's P values indicated. (**c**) Representative examples of PD-L1 expression for the three biopsies shown in Figure S1E. (**d,e**) Melanoma PD-L1 expression score for the 36 biopsies included in the study (d) stratified by therapy and patient response (e). Boxes show the interquartile range and the median, with Mann-Whitney's P values indicated. (**f**) B2M protein expression in pretreatment biopsies from 39 responders and 33 non-responders to anti-PD-1 monotherapy (Harel dataset, high-resolution liquid chromatography-mass spectrometry data after filtration for 70% valid values [1]). Mann-Whitney's P value is shown.

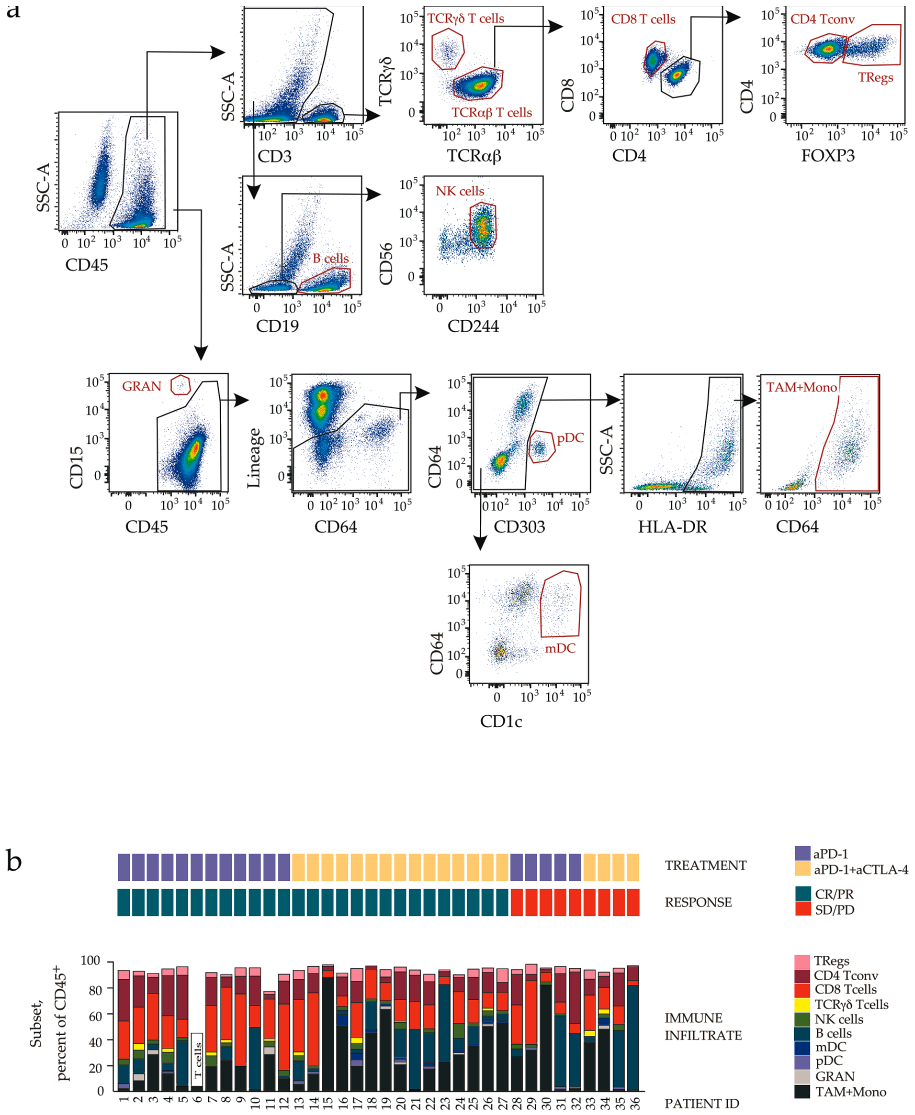

Figure S3. Immune contexture of individual melanoma biopsies. (**a**) Identification of tumor-infiltrating immune cells by flow cytometry. After gating CD45$^+$ events, lymphocytes were identified as follows (top row, left to right): SSC-A^{low}CD3$^+$ T-cells; TCRγδ$^+$ T-cells, and TCRαβ$^+$ T-cells further gated for CD8$^+$ T-cells, Tconv (conventional CD4$^+$FOXP3$^-$ T-cells) and TRegs (regulatory CD4$^+$FOXP3$^+$ T-cells); (second row): CD3-negatve events were gated for SSC-A^{low}CD19$^+$ B-cells, and SSC-A^{low}CD3^{neg}CD19^{neg} events were gated for CD244$^+$CD56$^+$ natural killer (NK) cells. (Third row, left to right): Alternatively, CD45$^+$ events were gated for CD15$^+$CD45^{int} granulocytes (GRAN). Lineage-negative events (CD3$^-$CD19$^-$CD56$^-$) were further gated for CD303$^+$ plasmacytoid dendritic cells (pDC), HLA-DR$^+$CD1c$^+$ myeloid dendritic cells (mDC) or HLA-DR$^{+/int}$CD64$^+$SSC-A$^{high/int}$ tumor-associated macrophages and monocytes (TAM+Mono). Final gates for individual subsets are shown in red; general gating strategy (Figure S1a) was applied prior to gating. (**b**) Cellular composition of tumor microenvironment in all pretreatment biopsies shown as a fraction of CD45$^+$ cells. Limited analysis was performed for patient 6 due to insufficient material.

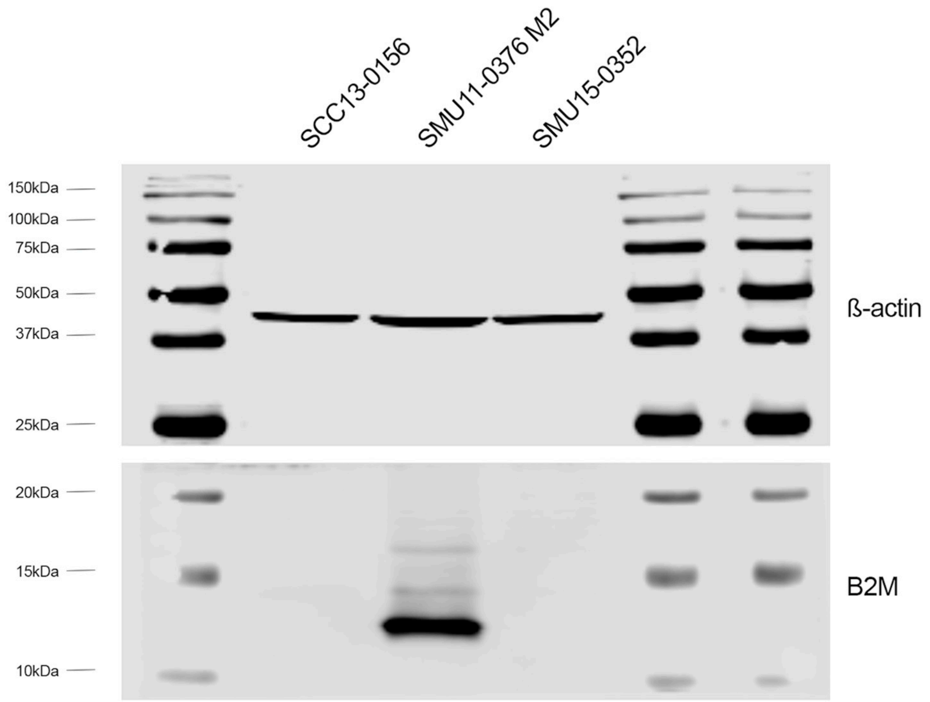

Figure S4. Uncropped images for Western blot gels used in Figure 1d.

Table S1. Antibodies and reagents used for flow cytometry.

Marker	Fluorescence	Company	Catalogue
CD1c/BDCA-1	PECy7	BioLegend	331516
CD3	BUV737	BD Biosciences	564308
CD3	BV786	BD Biosciences	565491
CD4	FITC	BioLegend	344604
CD4	AF700	BioLegend	357418
CD8	V500	BD Biosciences	561617
CD14	V500	BD Biosciences	561392
CD15	BV786	BD Biosciences	741013
CD16	AF700	BD Biosciences	557920
CD19	BUV737	BD Biosciences	564304
CD56	PE	Miltenyi Biotech	130–113–874
CD56	BUV737	BD Biosciences	564448
CD64	PE	BioLegend	305007
CD45	BV711	BioLegend	304050
CD45	BUV395	BD Biosciences	563792
CD45RO	BUV395	BD Biosciences	564291
CD45RA	BUV737	BD Biosciences	564442
CD134/OX40	PE-Cy7	BD Biosciences	563663
CD137/4-1BB	PE-Dazzle 594	BioLegend	309826
CD141	PE-Dazzle 594	BioLegend	344120
CD146/MCAM	PE-CF594	BD Horizon	564327
CD223/LAG3	PE	Miltenyi Biotech	130-105-452
CD244	PE-Cy7	BioLegend	329520
CD357/GITR	BV421	BD Biosciences	566423
CD271/NGFR	PE-Cy7	BioLegend	345110
CD273/PD-L2	APC	BioLegend	329608
CD274/PD-L1	BV421	BioLegend	329714
CD278/ICOS	APC	BioLegend	313510
CD279/PD1	BV421	BD Biosciences	562516
CD303	BV421	BD Biosciences	566427
CD366/TIM3	BV786	BD Biosciences	742857

EOMES	PE-Cy7	Thermo Fisher Scientific	25–4877–42
FC block		BD Biosciences	564220
FOXP3	PE-CF594	BD Biosciences	7235501
FOXP3	AF488	BioLegend	320212
Granzyme B	AF700	BD Biosciences	560213
HLA-A, B, C	AF700	BioLegend	311438
HLA-DR, DP, DQ	BUV395	BD Biociences	740302
KI67	APC	Thermo Fisher Scientific	17–5699–42
anti-human IgG4-Fc	PE	Southern Biotech	9200–09
Sox10	AF488	Santa Cruz	sc-365692 AF488
TBET	BV421	BD Biosciences	563318
TCR $\alpha\beta$	APC	BioLegend	306718
TCR $\gamma\delta$	BV421	BD Biosciences	744870
TIGIT/VSTM3	PE-Cy7	BioLegend	372714
LVE/DEAD Fixable	NIR	ThermoFisher Scientific	L10119

References

1. Harel M., Ortenberg R., Varanasi S.K., Mangalhara K.C., Mardamshina M., Markovits E., *et al.* Proteomics of Melanoma Response to Immunotherapy Reveals Mitochondrial Dependence. *Cell* **2019**; *179*: 236–250 e18.
